# Reconsidering the Role of Exocrine Pancreatic Insufficiency in Functional Dyspepsia

**DOI:** 10.5152/tjg.2025.25532

**Published:** 2025-10-10

**Authors:** Serdar Akca

**Affiliations:** Department of Gastroenterology, Antalya Training and Research Hospital, Antalya, Türkiye

Dear Editor,

I read with great interest the recent article by Kemik et al^1^ entitled “The Role of Pancreatic Enzyme Insufficiency in the Etiology of Functional Dyspepsia Resistant to Standard Treatment.” The topic is highly relevant, as exocrine pancreatic insufficiency (EPI) is increasingly recognized as an underdiagnosed and underestimated condition in the general population. Recent reviews highlight that the prevalence of EPI may reach 10%-20% in otherwise unselected populations, far exceeding rates seen in many co-conditions.^2^ Therefore, studies exploring the link between EPI and functional dyspepsia (FD) are of considerable clinical importance.

However, several methodological concerns should be noted. First, the analysis is based on a relatively small cohort, with multiple subgroup comparisons performed without adjustment for multiplicity, substantially increasing the risk of Type I error, particularly when several p-values are marginal.^3^ The absence of effect sizes and confidence intervals further limits the robustness of the findings.

Second, the inclusion of patients with diabetes mellitus (DM) in the FD group is problematic. The EPI is well documented in DM, and pancreatic function should be assessed and EPI excluded before classifying such patients as FD; otherwise, diagnostic overlap may confound the results.^4^^,^^5^

Finally, clarification is needed regarding patients with *Helicobacter pylori *(*Hp*). According to the Rome IV criteria, FD should only be diagnosed after exclusion of organic causes. Active *Hp* infection is considered *Hp*-associated dyspepsia, and FD can only be diagnosed after eradication therapy and persistence of symptoms.^6^^,^^7^

In conclusion, while the study underscores the potential role of EPI in patients with FD, its methodological limitations necessitate cautious interpretation. Importantly, these findings highlight the need for a high index of suspicion for EPI in FD patients. Future research should prioritize population-based studies to better define the prevalence of EPI among FD patients and to develop clearer diagnostic pathways for those presenting with dyspeptic symptoms.

## Data Availability

The data that support the findings of this study are available on request from the corresponding author.
